# Conference Report: WORKSHOP ON ASSESSMENT OF TESTING METHODOLOGY FOR GLASS, GLASS-CERAMIC, AND CERAMIC MATRIX COMPOSITES Gaithersburg, MD February 8, 1990

**DOI:** 10.6028/jres.096.026

**Published:** 1991

**Authors:** David C. Cranmer

**Affiliations:** Ceramics Division, National Institute of Standards and Technology, Gaithersburg, MD 20899

A workshop on mechanical testing methodology for glass, glass-ceramic, and ceramic matrix composites was held at NIST on February 8, 1990. The purpose of the workshop was to assess room- and elevated-temperature measurement techniques for whisker- and continuous-fiber reinforced ceramic composites and their constituents. Techniques being used for metal- and polymer-matrix composites as well as those developed exclusively for use with ceramic composites were discussed. Important points included the use of tensile tests, not flexure, for strength and creep measurements; the need for different tests for material development and for system design; the need to precisely control test parameters; and the importance of specimen preparation. Additional conclusions were that the same tests used for monolithic ceramics can be used for whisker-reinforced materials with few or no changes, and that interlaboratory round robin tests were needed to determine both the reproducibility and limitations of the methodologies as well as material behavior. Additional research areas identified included long-term deformation behavior of composites, environmental effects on material behavior, and the relation of constituent properties to composite properties.

## 1. Introduction

This paper summarizes a workshop on testing methodology for glass, glass-ceramic and ceramic matrix composites held at the National Institute of Standards and Technology on February 8, 1990. For brevity, the term “ceramic” refers to glass, glass-ceramic, and ceramic materials. The purpose of the workshop was to review and discuss mechanical property measurement techniques for whisker-and continuous-fiber reinforced ceramic composites and their constituents. A series of nine papers was presented detailing various aspects of mechanical testing of these materials. More details for each paper can be found in reference [1]. Prior to discussing the workshop, it is appropriate to review several salient features of composites testing. First, testing of polymer and metal matrix composites has a long history compared to ceramic matrix composites. Some of the test methods used for metal and polymer composites can be directly applied to the testing of ceramic matrix materials, while others can be adapted for use at elevated temperatures. One group of presentations was, therefore, selected to provide a background of standard and commonly used tests for evaluating polymer and metal matrix composites. A second group of presentations related the government and industrial experience with fracture of fiber-reinforced ceramics, primarily at room temperature. Finally, since there is little knowledge of the long-term deformation characteristics of these composite materials [2], a third group of presentations was selected to address long-term deformation.

The expectations for the workshop were fourfold. First, that appropriate measurement techniques suitable or adaptable for determination of the properties of the composites would be identified. Second, that appropriate measurement techniques suitable or adaptable for determination of the properties of the components (i.e., fiber, whisker, interface, and matrix) of the composites would be identified. Third, that areas where additional research and development were required to meet the needs for appropriate measurement techniques would be identified. Fourth, that techniques would be identified which are or can be made suitable as standard measurement methods. As will be shown below, each of these objectives were met. The following sections give synopses of the presentations and discussion for the various areas of composite testing.

## 2. Available Test Methods for Polymer and Metal Matrix Composites

A number of test methods have been used to evaluate the mechanical, thermal, and physical properties of polymer and metal matrix composites. The properties of interest are ultimate strength, yield strength, elastic moduli, strain to failure, fatigue life, creep, thermal expansion, thermal conductivity, thermal diffusivity, electrical conductivity, dielectric constant, and chemical resistance. The mechanical tests are almost exclusively destructive, while the physical tests can be either destructive or non-destructive. There are ASTM standard tests [3] for fiber density (D-276, D-792, D-1505, D-3800), fiber tensile properties (D-3379, D-3544, D-4018), composite tensile strength and modulus (D-638, D-3039), composite compressive strength and modulus (D-695, D-3410), flexural strength and modulus (D-790), composite shear strength and modulus (D-2344, D-3518, D-4255, E-143), composite tensile fatigue (D-3479), dielectric constant and dissipation factor (D-150), and dielectric strength (D-149). In addition, there are commonly used tests for shear strength and modulus such as the Arcan [4] and Iosipescu [5] methods.

## 3. Tensile Testing for Strength of Ceramic Matrix Composites

**Figure 1:**
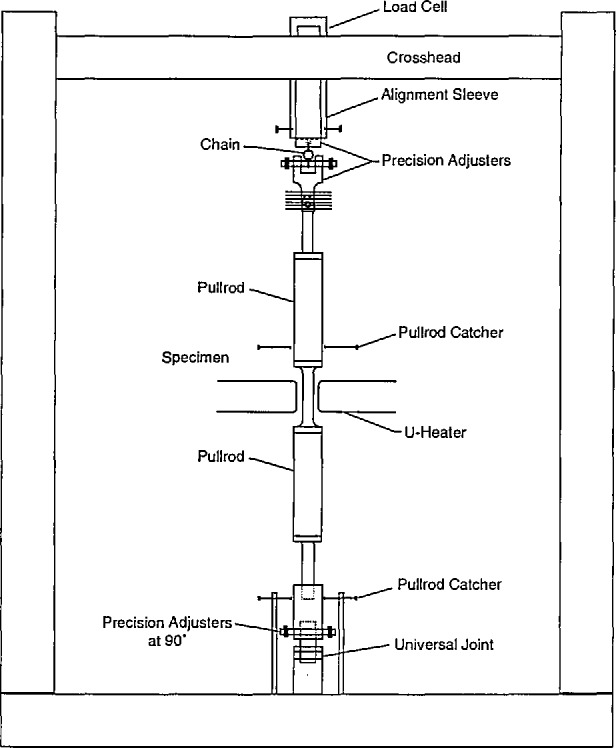
Schematic of tensile strength test apparatus using flexible load train.

There are several significant issues to be considered in designing a tensile strength test, including specimen design, specimen grip-ping arrangements, load train design, heating method, temperature measurement, and strain measurement. All the issues must be addressed properly to obtain a valid tensile test result. Failure to correctly implement them in the test will result in useless numbers for tensile strength. Additional details of the tensile test are in reference [6]. One viable tensile test uses a dogbone-shaped specimen containing three holes in the tab end. The three holes used to pin the specimen in the grips are the means by which the load is applied to the gage section. The remainder of the load train consists of a load cell, pull-rods, universal joints and a chain to control alignment and prevent introduction of bending moments into the system (fig. 1).

Another tensile test configuration which works well utilizes a rigid grip system (see reference [1]). Rigid grips have the advantages of staying aligned during the test; no “pre-load” is required to remove any slack in the load train; test specimens can be consistently and correctly mounted using simple tools such as calipers and depth gages; the rigid load train provides a stable platform for instrumentation (e.g., extensometer cables, thermocouples); and the degree of bending introduced into the specimen remains nearly constant throughout the complete load range. The specimen remains nearly constant throughout the complete load range. The specimen geometry can be straight-sided or dogbone-shaped, and can be tabbed or untabbed. Specific specimen dimensions depend on the test geometry, test conditions, and material availability. The tabbed, straight-sided specimen is the only one suitable for use with a unidirectionally reinforced composite with this configuration. Other specimens must use cross-plied material.

A third configuration discussed in more detail below applies the load to the shoulders of the tab section. This configuration uses dogbone-shaped specimens, and requires very tight tolerances on both the specimen and the loading fixture. Depending on the testing requirements, this configuration can be operated with either hot or cold grips.

Specimen preparation is a critical part of specimen design. Machining can have a significant impact on the behavior of the material, particularly where fibers are not parallel to the tensile axis. Improper grinding can lead to broken, debonded, and/or improperly aligned fibers during the test, resulting in inaccurate or incorrect numbers for the tensile strength. The degree of inaccuracy will also depend on fiber alignment (layup), fiber volume fraction, and relation of the tensile stress axis to the fiber orientation.

Strain can be measured using strain gages, laser sensors, clip-on extensometers, or high temperature rod extensometers. The choice of strain measurement tool depends on the specimen geometry, and the test geometry and conditions, as well as the degree of precision desired. Time can also be a significant factor in deciding on the precision required in the strain measurement. In practice, for strength measurements, the clip-on extensometers and laser dimension sensors do not have the required precision, and attention should be focussed on the high temperature rod extensometers. The contact point on the specimen for the rod extensometers is a critical area. Three methods can be used to ensure that the specimen deformation is transferred to the contact rods: pin holes, grooves in the material, and grooves in a paint overcoat. All three work satisfactorily under some conditions, but the choice depends on the specimen and the temperature of the test. Since introduction of bending moment is a concern, strain should be measured on more than one side of the specimen. This is necessitated by the fact that the specimens are heterogeneous and may be bent or warped as a result of the manufacturing or fabrication process.

There have been no studies yet which compare the same material using all three of the configurations mentioned above. While all three can be expected to provide good results, an interlaboratory comparison of at least two continuous fiber-reinforced composites is needed to discern testing reproducibility and limitations, as well as material behavior including reproducibility. Testing laboratories and suitable materials remain to be identified.

## 4. Tensile Testing for Creep of Ceramic Matrix Composites

Evaluation of creep and creep rupture of ceramic matrix composites is a relatively recent development, as most researchers and material developers have concentrated their efforts on more critical fabrication and strength issues. However, as the materials evolve, there will be a greater need for information about the long-term deformation characteristics including fatigue, creep and slow (or subcriticai) crack growth. Several tensile tests are available with which to evaluate the properties of ceramic composites as well as monolithic ceramics.

**Figure 2:**
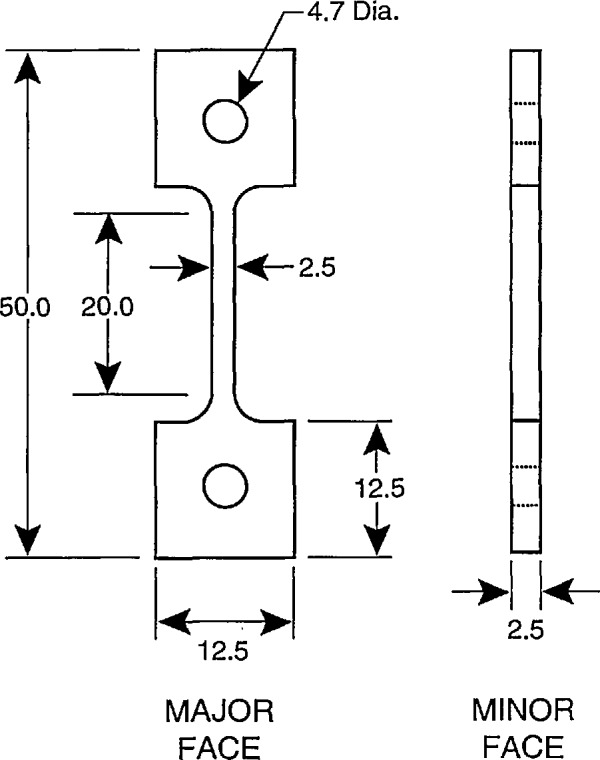
Typical dogbone specimen geometry used for tensile creep testing. Dimensions are in mm. The holes in the specimen are tapered to minimize bending strains.

A typical tensile creep test specimen and set-up are shown in figure 2. The holes in the tab sections of the specimen are tapered to minimize strains due to bending. The load train is similar to that described for the tensile strength test above in that it consists of loading rods to which the specimen is pinned. The loading rods are attached to universal joints, which in turn are connected to either the load cell (at the bottom) or to the air piston which provides the load (at the top). The entire specimen is located in the hot section of the furnace.

Changes in the gage length of the specimen are monitored via an optical telescope or a laser dimension sensor. Flags (with or without Pt wires attached) are fixed to the specimen to provide the measure of the gage length. A laser dimension sensor can provide an accuracy of ± 2 µm and acquires much more data than the optical telescope method. This degree of accuracy and data acquisition ability means that all three stages of creep can be monitored continuously, resulting in better measurements of rupture life, and hence better predictions of component reliability and lifetime.

Another test geometry for measuring tensile creep and strength has been developed at Oak Ridge National Laboratory [7]. The specimen has a cylindrical geometry with a machined buttonhead end for gripping. The shape is simple but requires very precise tolerances which are achievable with computer numerical controlled (CNC) grinding equipment. The technique has several advantages: symmetrical loading on the specimen, a simple gripping arrangement, relatively uniform load transfer which minimizes the bending moment on the specimen, and the ability to achieve large volume-to-surface area ratios. There are, however, at least two difficulties with this specimen: the cost associated with machining of the material, and the large amount of material required for each specimen. This test can be used to obtain design data, but, given the machining requirements, it may not be appropriate for fiber reinforced materials because of fiber breakage and misalignment.

**Figure 3:**
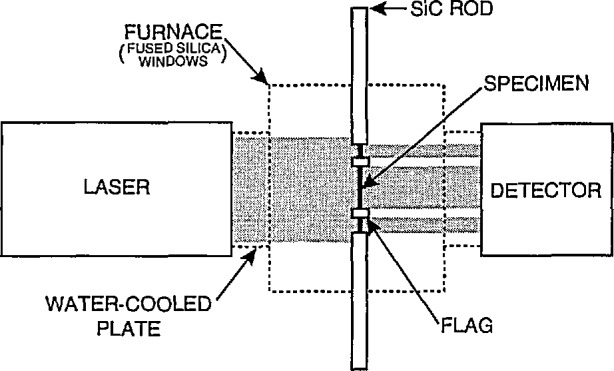
Schematic of tensile creep apparatus.

A different tensile creep (and fatigue) test [8] for ceramic matrix composites has also been developed. The apparatus is similar to that used for the shoulder-loaded tensile strength test described in the previous section. The grips can be either ceramic (e.g., SiC) for high temperature use, or superalloy for intermediate temperatures. A schematic of the gripping arrangement is shown in figure 3. Front-to-back alignment in the fixture is controlled by precision-machined inserts which are fastened to the body of the fixture after the specimen is in place. This configuration has been used to evaluate tensile strength, tensile creep, and tension-tension fatigue of fiber-reinforced ceramic composites.

The experimental data must be accompanied by proper microstructural evaluation to more fully understand the strength, creep, and fatigue failure mechanisms, and be able to make changes to the microstructure to enhance the desired properties while minimizing degradation of the remaining ones. As an example, for creep, in the case of a SiC_w_-Si_3_N_4_[9], it was found that the initial transient was dominated by devitrification of a glassy interfa-cial phase, and that cavitation at the SiC_w_/Si_3_N_4_ interface enhances the creep rate, thus reducing the composite’s lifetime. Compared to an unrein-forced Si_3_N_4_, there was no increase in creep resistance as a result of introducing the whiskers. This unexpected result is explained by cavitation which occurs at the SiC_w_/Si_3_N_4_ interface, and may possibly be altered by changing the initial glassy phase composition or content, or by appropriate surface treatment of the whiskers to minimize cavitation.

## 5. Determination of Fiber/Matrix Interfacial Properties

The strength of the fiber-matrix interface is one of the key parameters responsible for the stress-strain behavior and damage tolerance of ceramic composites. Two different types of tests are available to measure the fiber/matrix interfacial properties in fiber-reinforced ceramic composites. The first is based on an indentation technique to either push the individual fiber into the matrix or push the fiber through the matrix. The second relies on pulling a single fiber out of a matrix. These methods have been compared [10] to one another for a glass matrix material, and show similar results.

**Figure 4:**
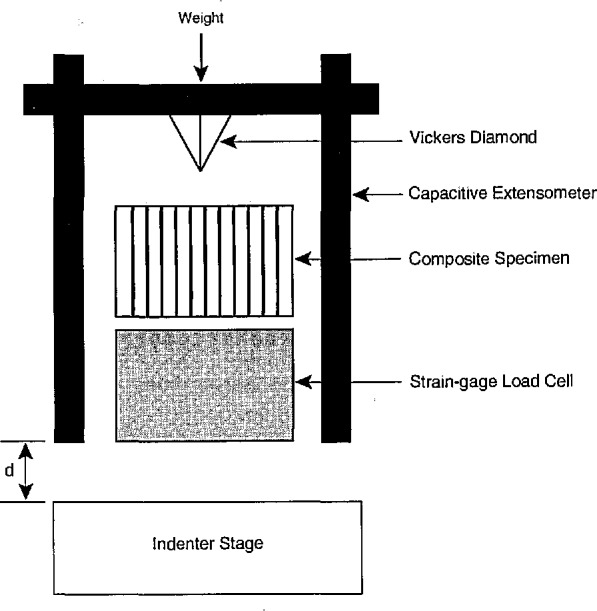
Schematic of instrumented indentation apparatus.

The indentation tests were performed using an instrumented indenter, allowing for independent determinations of force and displacement during the complete loading and unloading cycle. A schematic of the test apparatus is shown in figure 4. Displacement was determined using a pair of capacitance probes; the change in capacitance in a probe varies as it approaches a fixed target. Targets were fixed with respect to the specimen surface, and each probe was initially calibrated using a laser interferometer. Specimens for which indentation results have been obtained include 1 and 2 mm thick multifilament SiC/glass-ceramic and 0.3 mm thick monofilament SiC/borosilicate glass. The samples were at least partially flat and polished so that the capacitance probes did not have to be adjusted frequently.

**Figure 5:**
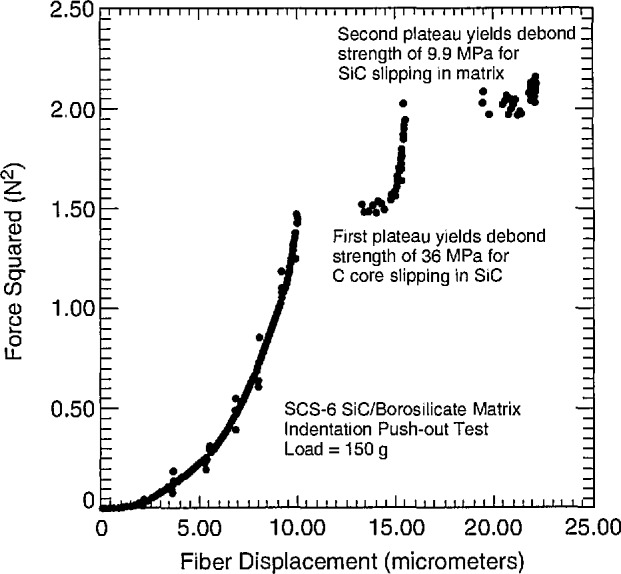
Force^2^-displacement curve from indentation test for SiC monofilament/borosilicate matrix composite. First plateau is for carbon core debonding from SiC, second plateau is for SiC debonding from borosilicate glass matrix.

For the SiC monofilament/borosilicate glass system, the push-out test can exhibit two plateaus in the (force)^2^-displacement curve (fig. 5). The force at the first plateau gives a value of *τ* of 36 MPa for the carbon core slipping in SiC and the force at the second plateau gives a value of *τ* of 10 MPa for the SiC slipping in the matrix. Tests on additional fibers gave an average value of *τ* of 30 ± 9 MPa for the core in SiC. The value of *τ* for SiC in the matrix is in reasonable agreement with *τ*_debond_ obtained from the single fiber pull-out test.

Examination of the SiC/glass-ceramic system shows that *τ* is dependent on the investigator as well as the technique [11]. The indentation push-in results yield *τ*’s varying from 1 to 10 MPa, and from 1 to 100 MPa, depending on the heat treatment. The discrepancy in heat treated materials is due to differences in the fiber-matrix interface bonding with some fibers being more tightly bound than others. This would lead to differences in both debond strength and frictional pull-out. The discrepancy in various untreated materials is due to both differences in the fiber-matrix bond and fiber misorientation with respect to the applied force.

The indentation tests use a minimal amount of material and can be performed on samples containing either large monofilaments or small diameter multifilament tows but provide information on only *τ*_friction_ (push-in) or *τ*_friction_ or *τ*_debond_ (push-Out), depending on indenter geometry and material characteristics. The push-out test can be performed at slower loading rates and with a different indenter geometry, thus allowing separation of the debonding strength from the interfacial friction stress in the force^2^-displacement curve. Preparation of push-out samples is more difficult than for push-in samples but the analysis is simpler and the results appear to be more reproducible. An additional potential advantage of the indentation method is that it may be adaptable for use as a quality assurance tool, since it can be used on small pieces of the as-fabricated composite. This application may not be realized until a clearer relationship is established between the debond strength/frictional shear stress and the macroscopic properties of the composite, such as strength.

## 6. Single Fiber Testing

There is a need for testing of the composite components, namely, the starting fibers, the matrix material, and the fiber/matrix interface. Test methods for determining the interface properties were discussed in the previous section. For the matrix material, recourse can be had to a large number of techniques developed over several decades. Several comprehensive reviews of these techniques have been published in the past 10–15 years [12, 13].

Determination of the properties of the starting fibers is a difficult task, particularly at elevated temperatures. Among the factors to be considered are testing of a single filament versus a multifilament yarn, test system compliance, gauge length, strain rate, grip material and pressure, strain measurement technique, and fiber diameter measurement. Each of these factors can significantly affect the measured result. As an example, when considering the measurement of a single fiber, it matters whether the fiber was indeed single or removed from a larger tow. For those removed from a multifilament tow, the fiber selection process itself may be expected to bias the results toward a higher strength, as fibers that break during removal from the tow would be discarded, not tested. This will affect not only the average strength, but any measures of strength distribution such as standard deviation and the Weibull modulus.

Room temperature testing can be accomplished using standard fiber tensile tests such as ASTM D-3379, D-3544, and D-4018. Elevated temperature testing provides a host of new considerations including furnace design, vertical versus horizontal testing, hot versus cold grips, grip materials, and temperature measurement and uniformity. The furnace design must consider chimney effects and the resultant difficulties associated with temperature stability and thermal gradients. An additional consideration is the effect of time at temperature, and the length of time required to thermally equilibrate the fiber prior to testing. The small diameter fibers typically are composed of small grains, which can grow rapidly when exposed to elevated temperatures. If it takes too long to perform the test, the material tested may no longer be representative of the initial fiber.

## 7. Fracture Toughness Determinations

One of the advantages of reinforced ceramics is that of increased damage tolerance. It has been shown [14] that inclusion of whisker reinforcement in a ceramic can result in as much as a four-fold increase in fracture toughness (*K*_Ic_). It can also result in an increase in fracture resistance with increasing crack length, known as R-curve behavior. In the case of whisker-reinforced materials, the R-curve is due to changes in fracture behavior as a result of crack interactions with the whiskers, wherein the whiskers bridge the crack behind the crack tip. Similar observations have been made in some monolithic ceramics such as alumina [15] except that whisker bridges behind the crack tip are replaced by grain bridges which exist as a result of the anisotropic microstructure.

The R-curve can be evaluated using an indentation-strength method wherein the strength of the composite is measured as a function of indentation load. The resulting curve is analyzed by assuming a power-law representation of the R-curve. This analysis in turn yields information about both the strength and toughness of the composite. When coupled with microstructural observations, it is then possible to determine the cause of the increase in toughness, and begin to make changes to the processing of the composite to take advantage of the bridging phenomenon and produce a more damage tolerant material. This may provide a useful method for developing a hybrid composite containing both whiskers and fibers, wherein the whiskers enhance the toughness of the matrix while the fibers provide the necessary directional tailoring of the composite properties.

## 8. Industrial Experience in Composites Testing

**Figure 6:**
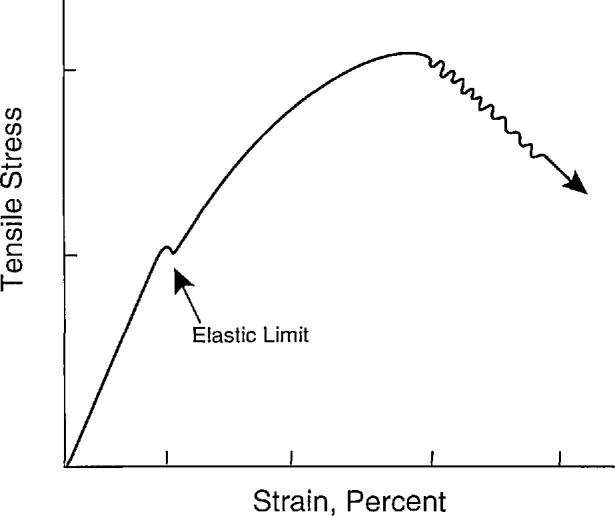
Typical stress-strain behavior of ceramic composite.

This section covers the experiences of two industrial firms, one a materials manufacturer, the other a materials user. One of the problems identified earlier is the need for a low cost, simple, reliable tensile test procedure for determining composite properties. Another problem is related to oxygen embrittlement. An example of this behavior is the reaction of carbon interfaces with environmental oxygen after first matrix microcracking has occurred. A number of currently available ceramic composites rely on a carbon or carbon-rich interface to obtain the debonding and sliding necessary to achieve the damage tolerance shown in figure 6. As shown there, a change in slope representative of first matrix microcracking is evident. At this point, the composite interface and fibers become exposed to the environment (generally including oxygen) and chemically react with that environment, thus adversely changing the properties of the interface and/or fiber. For example, the carbon layer can form CO or CO_2_, which outgasses from the interface. The underlying fiber (such as SiC) then can react to form an oxide which bonds tightly to the matrix, thus eliminating fiber sliding as a toughening mechanism.

Corning, Inc. has developed a simple test whereby the mechanical behavior of a representative composite can be characterized, and which complements the indentation tests described above. The method uses a simple rectangular parallelopiped (similar to a flexure bar), which is mounted in hydraulic wedge grips (as described in the tensile strength tests above) with metal foil shims. The mounted specimen is loaded until matrix microcracking is observed (precracking), then removed from the grips, exposed to the desired environmental conditions (temperature, atmosphere). The post-exposure specimen is reloaded in the grips and pulled to failure. This method has been applied to materials such as Corning’s calcium aluminosilicate (CAS) reinforced with Nicalon SiC and having a carbon fiber/matrix interface with the results showing a significant reduction in strength after exposure. When the interface is changed to a micaceous material, the post-exposure strength is much nearer to the pre-exposure strength, indicating an enhancement in properties due to the interface. The test has some limits at present, especially in that it has not been adapted for elevated temperature use, but for purposes of material development and the indication of trends in behavior, it is an acceptable test. Another limitation exists in the detection of initial microcracking of the matrix, which at present is detected using the deviation from linearity of the stress-strain curve. A more sophisticated approach would use acoustic emission, real-time microscopy, or other more sensitive techniques to determine the onset of microcracking. Additional cooperation is also required between the mechanical test experimenter and the chemical analysis person to ensure that the chemistry of the interface is known, both before and after exposure, in order to properly guide the changes required for improvements.

Areas which have not yet been addressed by the materials’ manufacturers are the effects of long term exposure (similar to the tests described above for creep) and the actual service geometries and conditions (rig tests). These are dealt with to a certain extent below.

The kind of information obtained on ceramic matrix composites by materials’ users can be dictated by external constraints, and have resulted in a collection of wisdom related to design practice such as MIL-HDBK-17 [16]. This handbook mandates requirements that must be met before a structure can be placed in service. The result is a series of design allowables. A typical requirement is that the strength of the material shall be such that it is sufficient to sustain the ultimate load without failure. A similar requirement for damage tolerance is that the structure shall be capable of performing its function in the presence of expected manufacturing and service induced damage.

Additional factors that must be taken into account are environmental effects (thermal as well as chemical), effects of defects, statistical variability of the material, long term behavior, and cyclic versus static loading effects. Assessment of these effects requires the user to conduct a large series of tests using multiple specimens. A typical series will examine a unidirectional material in tension in the 0, 90, and cross-ply directions; 0, 90, and cross-ply in compression; and 1–2, 1–3, and 2–3 shear at seven different temperatures ranging from −54 °C to the expected service temperature; creep rupture at elevated temperatures up to the expected service temperature; and fatigue at room and elevated temperature. This series of tests requires as many as 405 specimens.

Generic test method requirements to meet the testing needs cited above are that 1) the test be appropriate for the materials of interest, 2) the test be uniform and repeatable, and have a stable hot section, 3) the strain be measured accurately and precisely, 4) the test method be easy and repeatable, 5) the test be cost effective and have a reasonable turnaround time, and 6) the test efficiently use the available material. In attempting to meet these generic requirements, General Electric’s experience has resulted in use of number of test methods for determining the necessary properties. These include ASTM D-638 and D-3039 test methods, as well as the tensile test methods developed by Southern Research Institute and the Cortest to measure tensile strength; ASTM D-695 and D-3410 test methods to measure compressive strength; ASTM D-3518 and D-3518(C) test methods as well as an asymmetric four-point bend (AFPB) test (a variation of the Iosipescu test) and saddle geometries to determine in-plane shear strength; and ASTM D-3846 and D-2344 test methods as well as the AFPB to measure interlaminar shear strength. The Cortest for tension relies on shoulder loading of a dogbone-type specimen, as contrasted to the tab-pinned geometries of the other tensile tests.

A number of future testing needs to obtain basic properties and general test requirements were also identified. For basic properties, the desired information includes flatwise tension, flatwise compression, interlaminar shear modulus, long cycle fatigue, and modes I and II interlaminar fracture toughness. The general test requirements include the need for interlaboratory standardization and vendor qualification, a notched tension/compression/long cycle fatigue test, a biaxial test method, a pin bearing test, characterization of joints, and residual properties as a result of impact, erosion, and wear.

## 9. Summary and Conclusions

A number of techniques are available for measurement of the mechanical properties of ceramic matrix composites. An assessment was made of techniques for measuring strength, creep, and fatigue. For whisker-reinforced materials, test techniques can be the same as those used for monolithic ceramics. For continuous fiber-reinforced composites, specialized tensile test techniques must be used. There is a need for different tests for material development and system design, a need to control test parameters including temperature, and temperature and stress gradients, and a need to properly prepare specimens for testing. Flexural testing generally is inappropriate for determining the mechanical properties of the fiber-reinforced composites. There is also a need to develop standard tests and reporting information in order to facilitate comparison of data from one laboratory to another. Toward that end, an interlaboratory comparison of continuous fiber-reinforced composites is needed to determine reproducibility and limitations of the methodology, as well as material behavior.

Additional research areas were identified including long term (> 1000 h) deformation behavior of all of these materials (whisker- and fiber-reinforced), the changes in material behavior due to the test and/or service environment, and the relation of subcomponent tests (e.g., on fibers) to the composite properties especially in view of the fact that rule of mixtures does not adequately predict properties of these materials.
